# Supplementary material for: Solute carrier family 2 member 2 (glucose transporter 2): a common factor of hepatocyte and hepatocellular carcinoma differentiation
Source: PLoS One. 2025 Apr 25;20(4):e0321020. doi: 10.1371/journal.pone.0321020 (PMC12026939; doi:10.1371/journal.pone.0321020)
Supplement: S1 Fig — GLUT2 protein expression was quantified compared to the control cells., ** p < 0.01, and *** p < 0.001. (PPTX) [file pone.0321020.s001.pptx]

## Slide 1
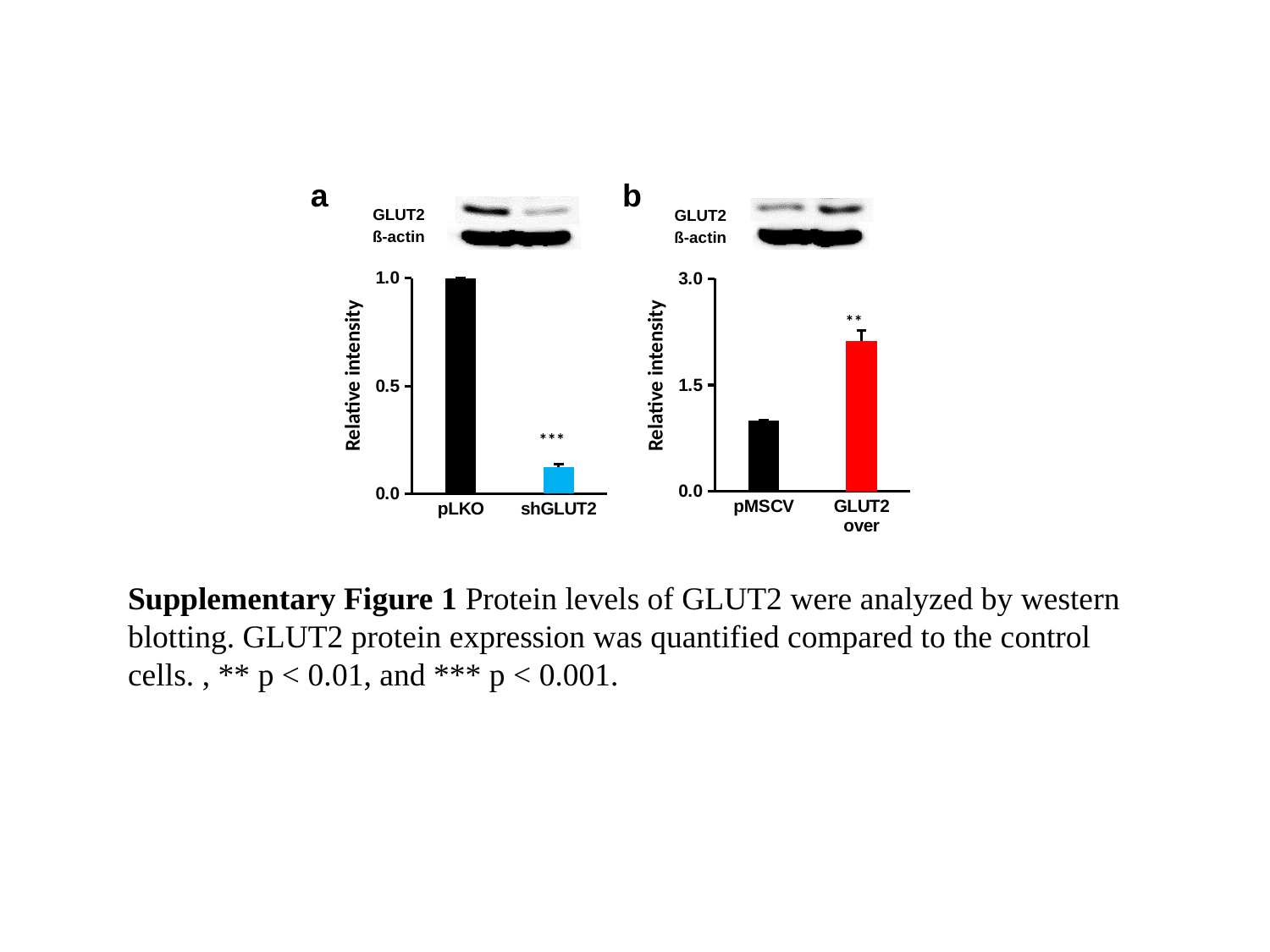

a
b
GLUT2
GLUT2
ß-actin
ß-actin
### Chart
| Category | |
|---|---|
| pLKO | 1.0 |
| shGLUT2 | 0.12309481455580147 |
### Chart
| Category | |
|---|---|
| pMSCV | 1.0 |
| GLUT2 over | 2.1159161444267647 |**
Relative intensity
Relative intensity
***
Supplementary Figure 1 Protein levels of GLUT2 were analyzed by western blotting. GLUT2 protein expression was quantified compared to the control cells. , ** p < 0.01, and *** p < 0.001.

## Slide 2
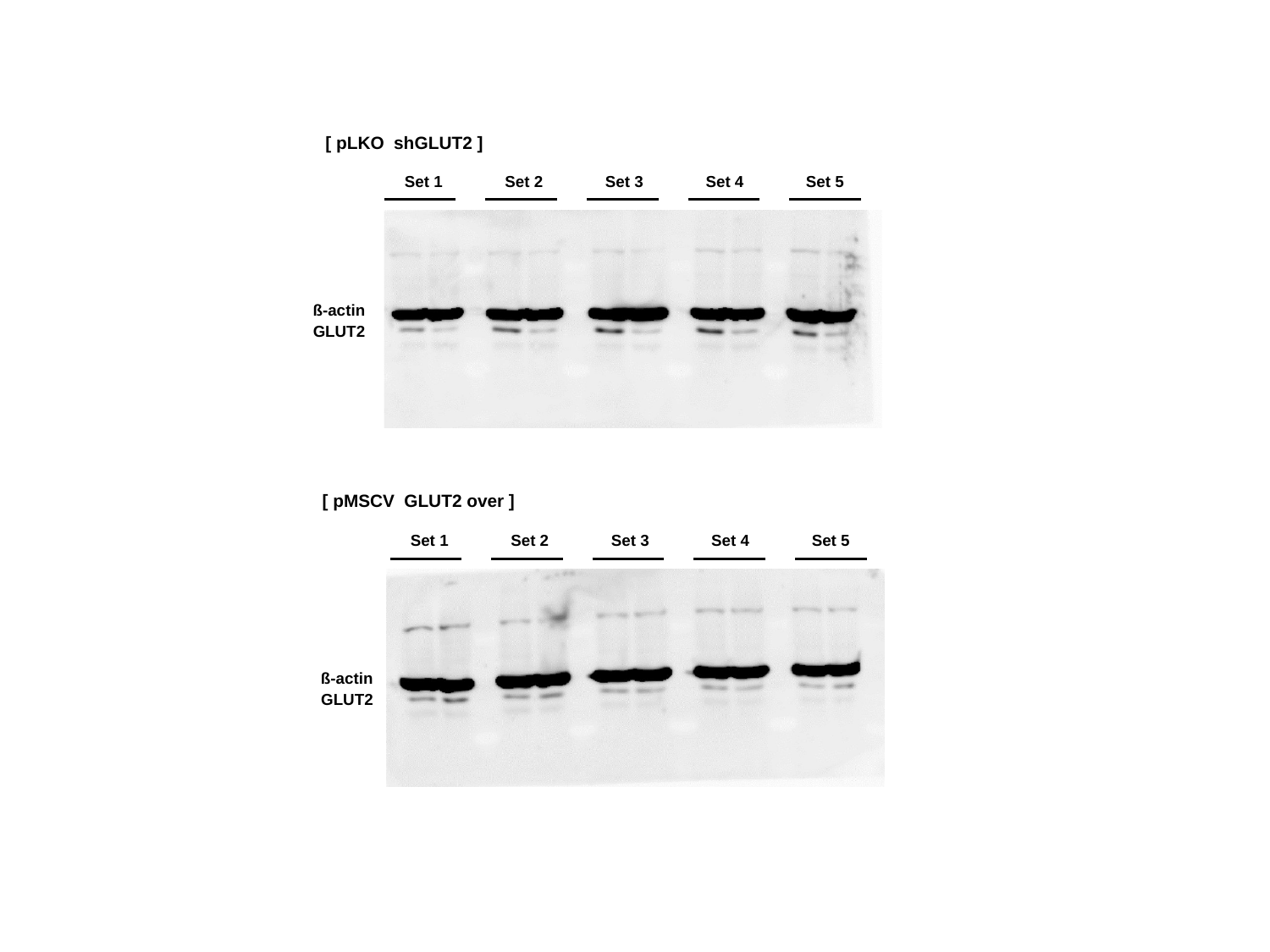

[ pLKO shGLUT2 ]
Set 1
Set 2
Set 3
Set 4
Set 5
ß-actin
GLUT2
[ pMSCV GLUT2 over ]
Set 1
Set 2
Set 3
Set 4
Set 5
ß-actin
GLUT2
