# Supplementary material for: Solute carrier family 2 member 2 (glucose transporter 2): a common factor of hepatocyte and hepatocellular carcinoma differentiation
Source: PLoS One. 2025 Apr 25;20(4):e0321020. doi: 10.1371/journal.pone.0321020 (PMC12026939; doi:10.1371/journal.pone.0321020)
Supplement: Attachment — Submitted filename: Point-to-Point Answers to Reviewers Comments.docx [file pone.0321020.s003.docx]

**Point-to-Point Answers to Reviewers Comments**

**Reviewer #1**

1.The introduction of the role of the SLC2A2 gene needs more description and literature support.

- Thank you for your kind comments. As you suggested, we have elaborated on the function of slc2a2 in the Introduction section to help readers better understand the manuscript. Below is the additional contents.

2.Source channels for zebrafish purchases need to be supplemented.

- As per your suggestion, we have made the necessary adjustments. In the Materials and Methods section, we have added that the zebrafish and fabp10a:mCherry transgenic zebrafish embryos used in this study were obtained from the Korea Zebrafish Resource Center (KZRC). Thank you for your valuable input.

3. The clarity of the images needs to be improved.

- We agree with your opinion. Accordingly, we have replaced the image with a higher-resolution version to enhance its clarity and quality.

4.After knocking out the target gene, the PCR results of other indicators did not change significantly.

- We agree with your opinion. To prevent confusion for the readers, we have revised the figure to display only the differentially expressed genes, removing the remaining genes from the figure.

5.The article has less experimental content, and it is recommended to add relevant protein experiments.

- As you suggested, additional experiments seem necessary. Therefore, we utilized the *fabp10a*:mCherry transgenic zebrafish embryos, which specifically mark the liver, to investigate the effects of slc2a2 knockdown (K/D). Our results showed that the liver size decreased when slc2a2 was knocked down, as confirmed at the protein level (Figure 4G). Additionally, to determine whether IGF1R protein levels also increase upon slc2a2 knockdown, we performed immunohistochemistry (IHC). The IHC results demonstrated that IGF1R expression increased across the whole body when slc2a2 was knocked down (Figure 5C).

**Reviewer 2**

**Major comments.**
1. To better establish whether Slc2a2 influences hepatocyte differentiation, it would be beneficial to analyze additional liver-specific markers. This could provide stronger evidence for the role of Slc2a2 in the differentiation process.

- Thank you for your kind suggestion. Based on your comments, we measured the mRNA level of g6pc1a.1, another mature liver marker, in zebrafish embryos. Additionally, we analyzed the mRNA levels of albumin, G6PC, and CYP3A4 using public data. Below are the experimental results:

**Figure 1A**

**Figure 4E**

2. The use of zebrafish embryos to explore liver differentiation is an excellent idea. As you have examined the relationship between EGFR, IGF1R, and slc2a2 in in vitro models, it would be interesting to investigate whether similar results are observed in the developing embryos.

- We agree with your opinion and have conducted experiments to investigate this relationship in zebrafish embryos. After performing a knockdown of slc2a2, we observed an increase in the mRNA level of IGF1R, as confirmed by qPCR. Additionally, IHC analysis revealed an increase in the protein level of IGF1R (Figure 5 B and C). These results suggest that slc2a2 may play a regulatory role in modulating IGF1R expression during liver differentiation in zebrafish embryos. Notably, we also examined EGFR expression but did not observe significant changes in either mRNA or protein levels following slc2a2 knockdown. Therefore, our analysis focused on IGF1R, as it demonstrated a more consistent and significant response. These findings further highlight the importance of IGF1R as a key regulatory pathway influenced by slc2a2 in liver differentiation.

3. In Figure 1, it would be helpful to check whether well-established liver differentiation markers (such as albumin, CYP3A4, G6PC) increase as the embryos develop. This would help confirm that the datasets from GEO accurately represent time points in embryonic development.

- Thank you for your kind comments. As per your suggestion, we analyzed the expression levels of albumin, G6PC, and CYP3A4 in datasets GSE132606, GSE25417, and GSE67848. The results demonstrated that the expression of all three genes increased as liver differentiation progressed (Figure 1A).

4. While the study has identified that SLC2A2 plays a role in liver differentiation and cancer progression, it does not fully elucidate the underlying mechanisms behind these effects. This lack of mechanistic clarity represents a limitation of the study. I suggest adding this point to the Discussion section of the manuscript to acknowledge this limitation.

- Thank you for your valuable suggestion. As you recommended, we have included this point in the Discussion section of the manuscript to acknowledge the limitation regarding the lack of mechanistic clarity behind the effects of SLC2A2 on liver differentiation and cancer progression. This addition highlights the need for further studies to fully elucidate the underlying mechanisms.
